# Magnolol induces cell death through PI3K/Akt‐mediated epigenetic modifications boosting treatment of *BRAF‐* and *NRAS*‐mutant melanoma

**DOI:** 10.1002/cam4.1978

**Published:** 2019-02-21

**Authors:** Abdullah Al Emran, Brinda Reddy Chinna Chowdary, Farzana Ahmed, Heinz Hammerlindl, Antje Huefner, Nikolas K. Haass, Wolfgang Schuehly, Helmut Schaider

**Affiliations:** ^1^ Dermatology Research Centre The University of Queensland Diamantina Institute, Translational Research Institute Brisbane Queensland Australia; ^2^ Centenary Institute of Cancer Medicine and Cell Biology Camperdown New South Wales Australia; ^3^ The University of Queensland, The University of Queensland Diamantina Institute, Translational Research Institute Brisbane Queensland Australia; ^4^ Department of Pharmaceutical Chemistry, Institute of Pharmaceutical Sciences University of Graz Graz Styria Austria; ^5^ Department of Pharmacognosy Karl‐Franzens University Graz Styria Austria

**Keywords:** Akt, BRAF, histone mark, *Magnolia officinalis*, magnolol, melanoma, NRAS, PI3K

## Abstract

Most *BRAF*‐mutant melanoma patients experience a fulminate relapse after several months of treatment with BRAF/MEK inhibitors. To improve therapeutic efficacy, natural plant‐derived compounds might be considered as potent additives. Here, we show that magnolol, a constituent of *Magnolia officinalis*, induced G1 arrest, apoptosis and cell death in *BRAF‐* and *NRAS‐*mutant melanoma cells at low concentration, with no effect in *BRAF‐* and *NRAS* wild‐type melanoma cells and human keratinocytes. This was confirmed in a 3D spheroid model. The apoptosis‐inducing effect of magnolol was completely rescued by activating Akt suggesting a mechanism relying primarily on Akt signaling. Magnolol significantly downregulated the PI3K/Akt pathway which led to a global decrease of the active histone mark H3K4me3. Alongside, the repressive histone mark H3K9me3 was increased as a response to DNA damage. Magnolol‐induced alterations of histone modifications are reversible upon activation of the Akt pathway. Magnolol‐induced a synergistic effect in combination with either BRAF/MEK inhibitors dabrafenib/trametinib or docetaxel at a lower concentration than usually applied in melanoma patients. Combination of magnolol with targeted therapy or chemotherapy also led to analogous effects on histone marks, which was rescued by Akt pathway activation. Our study revealed a novel epigenetic mechanism of magnolol‐induced cell death in melanoma. Magnolol might therefore be a clinically useful addition to BRAF/MEK inhibitors with enhanced efficacy delaying or preventing disease recurrence.

## 1 INTRODUCTION

Melanoma patients harbor BRAF mutations in 40%‐60% resulting in constitutive activation of prosurvival signaling through the MAPK pathway.^1^ Targeted therapies against BRAF have shown promising results and a profound effect with 80% overall response rate in melanoma patients harboring the BRAF^V600E^ mutation. However, at least 50% of the patients develop resistance after 6‐7 months of treatment.^2^ Therefore, recurrent resistance remains a major drawback of efficient melanoma treatment in these patients.

Natural products are a valuable resource for the development of therapeutics, and, in particular, plant‐derived alkaloids provided highly active cytotoxic lead structures. Many of the modern anticancer drugs are derived from plants for example docetaxel and paclitaxel (taxanes) from *Taxus brevifolia*, vincristine, and vinblastine from *Catharanthus roseus* or the chromone alkaloid flavopiridol from *Dysoxylum binectariferum*.^3^

The biphenyl neolignan magnolol is a major constituent obtained from the bark of the Chinese tree *Magnolia officinalis*. In the early 1990s, researchers found that magnolol decreases the concentration of hydroxyl radicals and inhibits lipid peroxidation in animal experiments.^4^ Recent studies revealed that magnolol exhibits various medicinal properties including antiproliferative, antioxidant, antiinflammatory,^5^ and anticancer^6^ effects. Magnolol, honokiol and its derivatives have also been shown to be potent GABA_A_ receptor agonists^7^ and inverse cannabinoid 2 receptor agonists.^8^ Additionally, honokiol activates Sirtuin‐3 (SIRT3, mitochondria‐dependent deacetylase) which can act as a tumor suppressor via decrease in ROS production and regulating HIF1.^9^ Along this, magnolol also plays an important role to decimate cancer cells by inducing apoptosis through increased production of caspases‐3, 8, and 9, suppression of Bcl‐2 expression and activation of death receptor and mitochondrial pathways.^10^ As melanoma patients experience most often a disease relapse during targeted therapies, plant‐derived lead structures may possess potential to be developed into a useful addition to existing therapies. Magnolol‐induced apoptosis has been studied in various cancer types including melanoma.^11^ As a result of these multiple beneficial effects of magnolol against various cancer types, the proposed application in clinical studies as an additional therapeutic agent is tempting. However, mechanisms of magnolol‐induced cell death in melanoma remain poorly understood.

Here, we show that magnolol‐induced cell death is mediated through downregulation of the PI3K/Akt pathway, which led to a decrease of the active histone mark H3K4me3 in melanoma cells which has not been reported earlier. Additionally, combinatorial treatment of low‐dose magnolol and targeted therapy or chemotherapy led to an increase in cell death in *BRAF‐* and *NRAS*‐mutant melanoma cells demonstrating a synergistic effect.

## 2 MATERIALS AND METHODS

Details of materials and methodology are provided as supporting information.

## 3 RESULTS

### 3.1 Magnolol and its analogue, 5,5'‐di‐(*tert*‐butyl)‐biphenyl‐2,2'‐diol, induce cell death in *BRAF‐* and *NRAS‐*mutant melanoma cells

**Figure 1:**
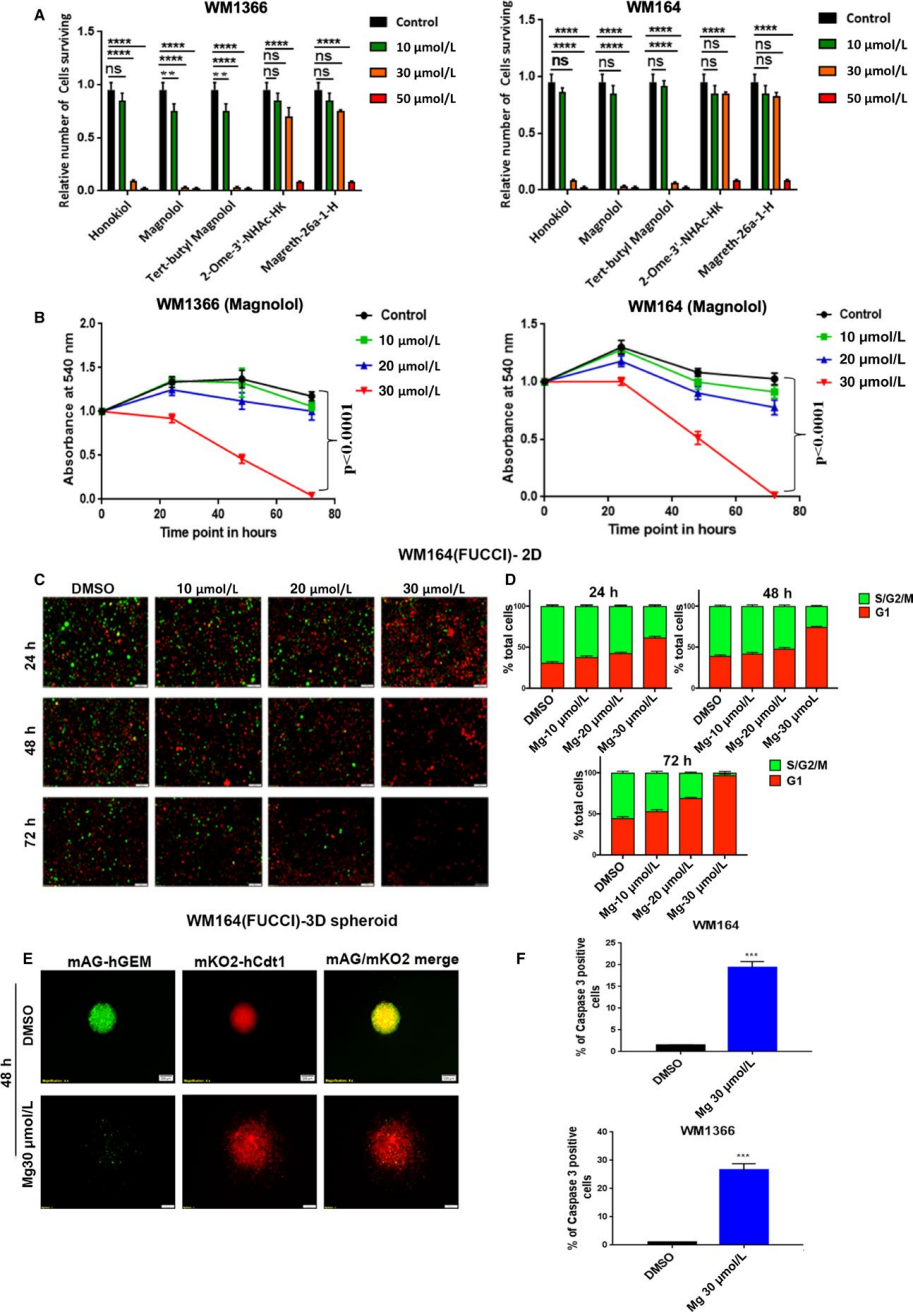
Magnolol induces cell death, growth arrest, and apoptosis in WM1366 (*NRAS*‐mutated) and WM164 (*BRAF*‐mutated) cell lines. (A) Quantitative analysis of cell survival. 1 × 10^5^ cells were plated in 24‐well plates and allowed to adhere for 24 h and then treated with respective drugs at indicated doses in triplicate. DMSO was used as control. After 72 h of treatment, cells were subjected to crystal violet staining. (B) Cell viability assessed by MTT assay. 5000 cells per well were plated on a 96‐well plate and incubated for 24 h. The following day, cells were treated with respective drugs at indicated doses in quadruplicates and viable cell numbers were determined at 24, 48, and 72 h. Absorbance was measured at a wavelength of 540 nm, normalized to time point 0. Statistical significance was calculated using an unpaired *t* test; ns not significant, *P ≤ 0.05, **P ≤ 0.01, ***P ≤ 0.001. (C) Epifluorescence microscopy images of FUCCI‐WM164 cells in 2D culture after treatment with magnolol with indicated concentrations and treatment periods. DMSO was used as control. Red, G1phase; yellow, early Sphase; green, late S/G2/M phase. (D) Quantification of the FUCCI red and green images by ImageJ, n = 2 independent experiments. (E) Epifluorescence microscopy images of FUCCI‐WM164 3D spheroids. A total of 50 000 FUCCI‐WM164 cells were seeded on solid agar in a 96‐well plate to form 3D spheroids. After three days, spheroids were treated with either DMSO or 30 µmol L^−1^ magnolol for 48 h. (F) WM164 and WM1366 cells were treated with either DMSO or 30 µmol L^−1^ magnolol for 60 h and analyzed for caspase‐3 positive cells by flow cytometry. Error bars represent the standard deviation of the mean. Statistical analysis was performed by a paired *t* test where ***denotes *P* < 0.001, ****P < 0.0001

Magnolol, honokiol, and derivates (Figure S1A) were first assessed for their efficacy in *NRAS*‐mutant WM1366 and *BRAF*‐mutant WM164 melanoma cells by crystal violet assay. Details of the compounds’ chemical structures, names, molecular weights, and numbers are shown in Table S1. Cells were treated at the indicated concentrations of the compounds for 72 hours. Magnolol, 5,5'‐di‐(*tert*‐butyl)‐biphenyl‐2,2'‐diol and honokiol were found to be very effective to kill *BRAF/NRAS*‐mutant melanoma cells at a concentration of 30 µmol L^−1^ in comparison with 2‐Ome‐3’‐NHAc‐HK and Magreth‐26a‐1‐H (Figure 1A, Figure S1B). As magnonol showed a slightly stronger activity than honokiol to kill melanoma cells at 30 µmol L^−1^, further studies were carried out with magnolol and its derivative 5,5'‐di‐(*tert*‐butyl)‐biphenyl‐2,2'‐diol (Figure S1C; from here on “tert‐butyl magnonol”). Cytotoxic activity of magnolol and tert‐butyl magnonol was assessed after 24, 48, and 72 hours by MTT assay. Time‐ and dose‐dependent cell death of melanoma cells was observed for both compounds (Figure 1B, Figure S1C). However, the cell death‐inducing effect by tert‐butyl magnonol was not found to be higher than that of magnolol; therefore, we continued to test magnolol alone. Along this line, magnolol‐induced cell death was not observed in the *BRAF/NRAS* wild‐type melanoma cell line, D24 and the human immortalized keratinocyte cell line, HaCaT (Figure S1D) suggesting that the effect of magnolol at lower concentrations might be specific for *BRAF/NRAS*‐mutant cancer cells.

### 3.2 Magnolol inhibits proliferation by inducing G1 arrest and apoptosis

To determine the effect of magnolol on the cell cycle in melanoma cell lines, a fluorescent ubiquitination‐based cell cycle indicator (FUCCI) system was used in which red fluorescence indicates G1, yellow early S and green S/G2/M phase.^12^ *BRAF*‐mutant FUCCI‐WM164 and FUCCI‐WM983B cell lines^13^ were used to determine the effect of magnolol at different stages of the cell cycle in real time. Cells were exposed to increasing concentrations of magnolol ranging from 0 to 30 µmol L^−1^ for 72 hours. Representative images of the cell cycle at different concentrations including control were captured within 24 hours by fluorescence microscopy. S/G2/M phase of the cell cycle (green) was evident for the lower concentrations at the early time points, but it decreased gradually with increasing concentrations and G1 arrest (red) was induced with increasing concentration from 24 to 48 hours implying that the magnolol‐induced effect on growth arrest occurred in a dose‐ and time‐dependent manner. It was notable that after 72 hours only a few G1 arrested (red) cells were found as most of the cells had died at this time point (Figure 1C‐D, Figure S1E‐F).

The effect of magnolol was recapitulated in a FUCCI‐WM164 3D spheroid model which mimics thein vivo tumor architecture and microenvironment more faithfully that 2D culture.^13^, ^14^ Like in 2D cultures, magnolol also induced G1 arrest in spheroids after 48 hours compared to DMSO control (Figure 1D). Next, we investigated apoptosis‐induced cell death of magnolol by a caspase‐3 assay. A significant portion of caspase‐3 positive cells was found in WM164 and WM1366 cells upon treatment with 30 µmol L^−1^ magnolol compared to control after 60 hours (*P* < 0.001) (Figure 1E, Figure S2A).

### 3.3 Magnolol impedes prosurvival signaling pathways

**Figure 2:**
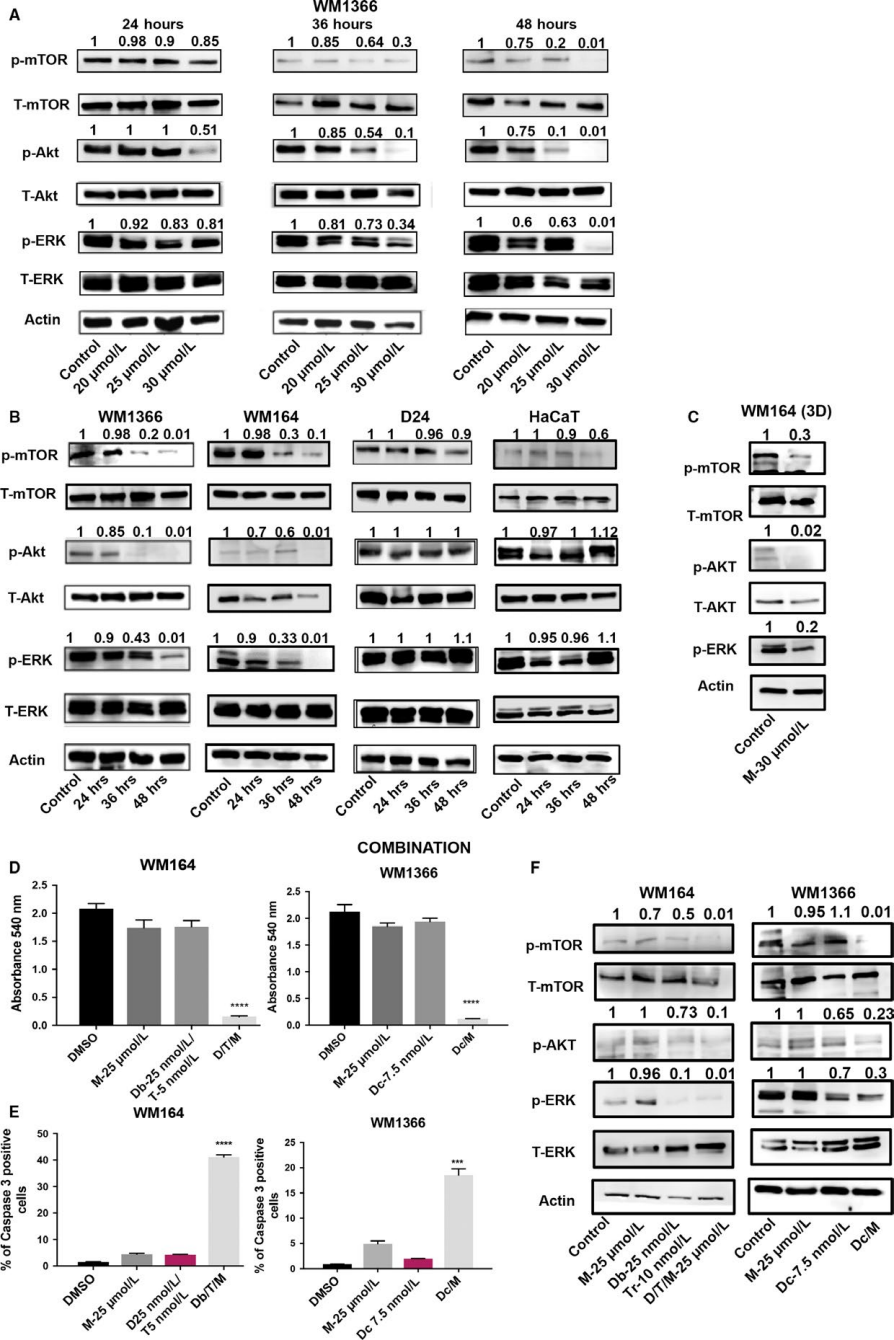
Magnolol downregulates the MAPK‐ERK and PI3K/Akt pathways either alone or in combination with targeted or chemotherapy in melanoma cells. (A) WM1366 cells were subjected to either DMSO or magnolol at indicated concentrations and treatment periods. Proteins were isolated and immunoblotted for p‐mTOR, t‐mTOR, p‐Akt, t‐Akt, p‐ERK, t‐ERK. Actin was used as a loading control. (B) In a separate set of experiments, WM1366, WM164, D24 and HaCaT cells were exposed to 30 µmol L^−1^ magnolol for indicated treatment periods. Proteins were isolated and immunoblotted for the above‐mentioned antibodies. (C) WM164 spheroids were exposed to DMSO or 30 µmol L^−1^ magnolol for 48 h. Proteins were immunoblotted for the antibodies mentioned in (A). (D) Cell viability assessed by MTT assay. WM164 cells were exposed to 25 µmol L^−1^ magnolol, 25 nmol L^−1^ dabrafenib/5 nmol L^−1^ trametinib or 25 nmol L^−1^ dabrafenib/5 nmol L^−1^ trametinib/25 µmol L^−1^ magnolol; WM1366 cells were exposed to 25 µmol L^−1^ magnolol, 7.5 nmol L^−1^ docetaxel or 7.5 nmol L^−1^ docetaxel/25 µmol L^−1^ magnolol for 72 h. DMSO was used as a control. Statistical significance was determined by the one‐way ANOVA test. (E) Caspase‐3 assay by flow cytometry. WM164 cells were subjected to either DMSO, 25 µmol L^−1^ magnolol, 25 nmol L^−1^ dabrafenib/5 nmol L^−1^ trametinib or 25 nmol L^−1^ dabrafenib/5 nmol L^−1^ trametinib/25 µmol L^−1^ magnolol; WM1366 cells were exposed to either DMSO, 25 µmol L^−1^ magnolol, 7.5 nmol L^−1^ docetaxel or 7.5 nmol L^−1^ docetaxel/25 µmol L^−1^ magnolol for 60 h. Statistical analysis was performed by the paired *t* test. Error bars indicate the standard deviation of the mean (n = 3, biological replicates). (F) WM164 and WM1366 cells were treated with the above‐mentioned concentration of drugs (E) for 48 h. Proteins were isolated and immunoblotted for p‐mTOR, t‐mTOR, p‐Akt, p‐ERK, t‐ERK. Actin was used as a loading control. All immunoblot were quantified by densitometry using ImageJ, and values were normalized to the loading control

Magnolol is known to inhibit various molecular signaling pathways as described in previous studies.^15^ Of these, the MAPK‐ERK and PI3K/Akt pathways are usually activated in cancer cells to promote cell survival.^16^ Hence, the effect of magnolol on these molecular signaling pathways was investigated. Magnolol downregulated phosphorylation levels of mTOR, Akt, and ERK in a time‐ and dose‐dependent manner in WM1366. The most profound effect was observed after 48 hours at 30 µmol L^−1^ magnolol (Figure 2A). Subsequently, 30 µmol L^−1^ magnolol was further tested on WM1366, WM164, D24, and HaCat cells. Phosphorylation of mTOR, Akt, and ERK was downregulated in WM1366 and WM164 cells after 48 hours. However, phosphorylation levels of these molecules remained relatively unchanged upon magnolol treatment in D24 and HaCaT cells which might explain why these cells were insensitive to these concentrations of magnolol (Figure 2B). A similar effect on downregulating signaling by magnolol was observed in the WM164 3D spheroid model (Figure 2C).

### 3.4 Magnolol induces a synergestic effect with molecular targeted therapies or chemotherapy to promote cell death in *BRAF/NRAS*‐mutant melanoma cells

Magnolol has been shown to inhibit the MAPK signaling pathway which prompted us to probe for combined BRAF/MEK inhibition with a low concentration of magnolol. Combined BRAFi/MEKi/magnolol was tested in WM164 cells. Cells were exposed to 25 µmol L^−1^ of magnolol in combination with 25 nmol L^−1^ dabrafenib (BRAF inhibitor) and 5 nmol L^−1^ trametinib (MEK inhibitor) and dabrafenib/trametinib or magnolol alone for 72 hours subjected to crystal violet staining. The combination of magnolol with dabrafenib and trametinib synergistically induced cell death at 72 hours (Figure S2B). A similar concentration of magnolol was employed with a low dose of the chemotherapeutic drug docetaxel (7.5 nmol L^−1^) in *NRAS*‐mutant WM1366 cells. Likewise, the combination of magnolol with docetaxel showed a potent effect on cell death (Figure S2B). Cell survival was further tested using an MTT assay and a significant cell death was observed for the combination of magnolol with targeted or chemotherapy (*P < *0.001) (Figure 2D). No significant cell death was observed upon exposure of *BRAF/NRAS* wild‐type D24 cells and HaCaT cells to magnolol and docetaxel indicating that *BRAF/NRAS* wild‐type cells might require a higher dosage of magnolol and chemotherapy than that of mutated cells (Figure S2C). A significant proportion of caspase‐3‐positive cells was identified upon exposure to magnolol/dabrafenib/tramentinib in WM164 cells and magnolol/docetaxel in WM1366 cells (*P* < 0.001, Figure 2E, Figure S2D). Further, the combination was tested for changes in prosurvival signaling cascades with a profound downregulation of p‐Akt, p‐mTOR, and p‐ERK compared to the single treatment in WM164. Analogous effects were observed for the combination of magnolol and docetaxel in WM1366 (Figure 2F).

### 3.5 The PI3K/Akt pathway is crucial for magnolol‐induced epigenetic modifications in *BRAF‐* and *NRAS*‐mutant melanoma cells

**Figure 3:**
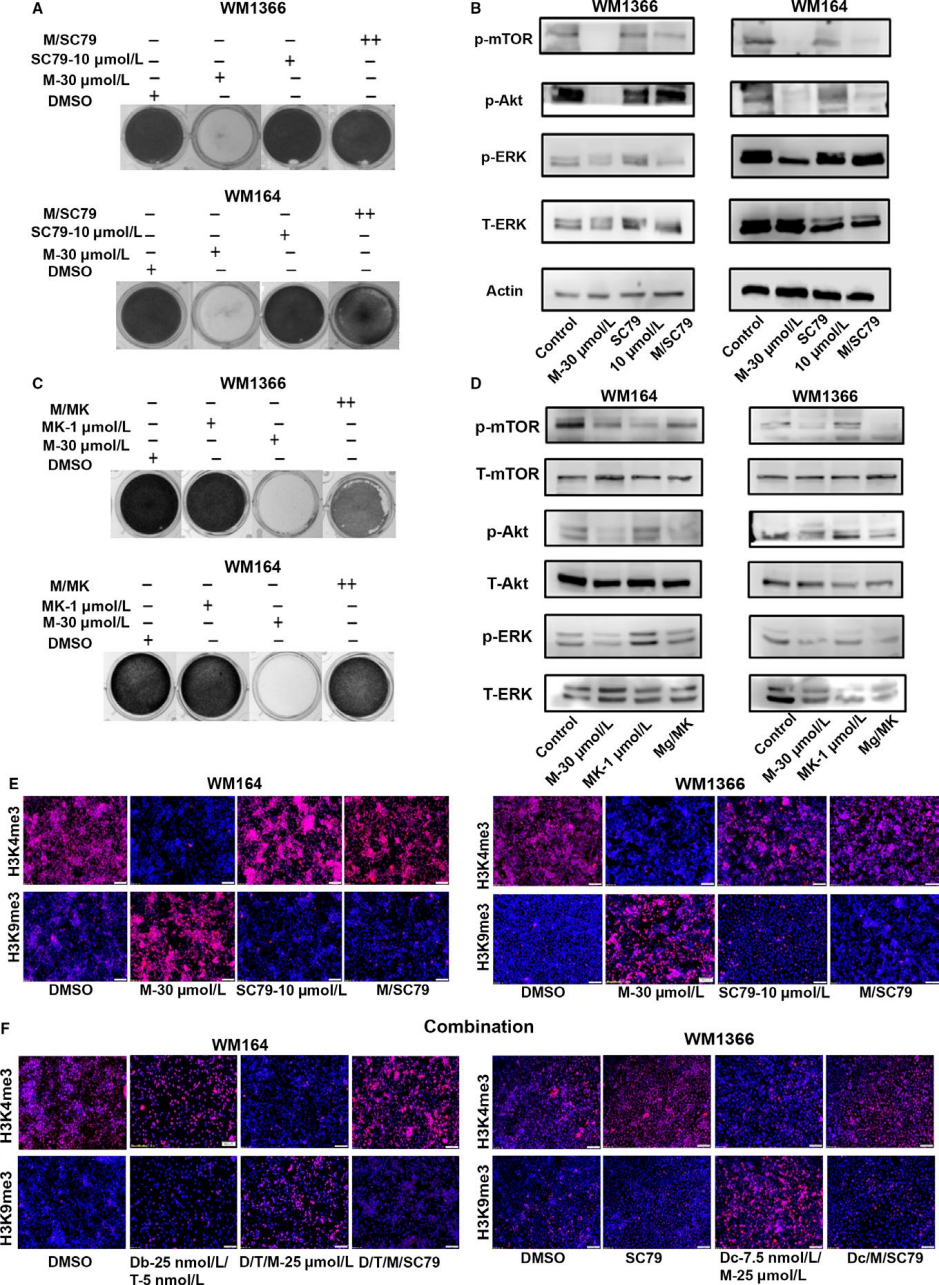
Akt pathway activation leads to survival, pathway reactivation, and histone remodeling of magnolol‐treated cells. (A) WM1366 and WM164 cells were exposed to DMSO, 30 µmol L^−1^ magnolol, 10 µmol L^−1^ SC79 or combination of 30 µmol L^−1^ magnolol/10 µmol L^−1^ SC79 for 72 h. Medium was aspirated following the treatment and cells fixed with 4% paraformaldehyde (PFA). Cells were stained with 0.5% crystal violet (n = 3, biological replicates). (B) In a separate experiment, WM1366 and WM164 cells were exposed to DMSO, 30 µmol L^−1^ magnolol, 10 µmol L^−1^ SC79 or a combination of 30 µmol L^−1^ magnolol/10 µmol L^−1^ SC79 for 48 h. Proteins were subjected to immunoblotting with p‐mTOR, p‐Akt, p‐ERK, t‐ERK. Actin was used as a loading control. (C) WM164 and WM1366 cells were treated with DMSO, 30 µmol L^−1^ magnolol, 1 µmol L^−1^ MK2206 or 30 µmol L^−1^ magnolol/1 µmol L^−1^ MK2206 for 72 h. Cells were stained with 0.5% crystal violet and fixed with 4% PFA. (D) In a separate experiment, WM164 and WM1366 cells were treated with the above‐mentioned drugs for 48 h. Proteins were isolated and immunoblotted with p‐mTOR, t‐mTOR, p‐Akt, t‐Akt, p‐ERK, t‐ERK. (E) WM1366 and WM164 cells were exposed to DMSO, 30 µmol L^−1^ magnolol, 10 µmol L^−1^ SC79 or combination of 30 µmol L^−1^ magnolol/10 µmol L^−1^ SC79 for 48 h in a 24‐well plate. Following treatment cells were fixed with 4% PFA and blocked with 0.3% TritonX100, 5% goat serum in 1% BSA and blotted for H3K4me3 and H3K9me3. Expression of these histone marks was determined by immunofluorescence. Representative merged images of DAPI (blue) and antibody (red) are shown (10X magnification). (F) In a separate experiment, WM164 cells were exposed to DMSO, 25 nmol L^−1^ dabrafenib/5 nmol L^−1^ trametinib, 25 nmol L^−1^ dabrafenib/5 nmol L^−1^ trametinib/25 µmol L^−1^ magnolol or 25 nmol L^−1^ dabrafenib/5 nmol L^−1^ trametinib/25 µmol L^−1^ magnolol/10 µmol L^−1^ SC79. WM1366 cells were exposed to DMSO, 10 µmol L^−1^ SC79, 7.5 nmol L^−1^ docetaxel/25 µmol L^−1^ magnolol or 7.5 nmol L^−1^ docetaxel/25 µmol L^−1^ magnolol/10 µmol L^−1^ SC79 for 48 h. Similarly, these cells were stained for H3K4me3 and H3K9me3 by immunofluorescence

Previous studies suggested that magnolol downregulates the PI3K/Akt pathway along with inhibition of other pathways.^17^ However, the mechanisms of PI3K/Akt‐mediated cell death upon magnolol treatment remained elusive. To determine the mechanism of action of magnolol, the Akt pathway was activated by a small molecule activator, SC79 specific for p‐Akt. Interestingly, activation of the Akt pathway rescued the effect of magnolol in *BRAF‐* and *NRAS*‐mutant melanoma cells suggesting magnolol might primarily be acting through the PI3K/Akt pathway (Figure 3A). Indeed, the rescue effect of magnolol upon Akt activation was mediated through increased p‐Akt, p‐mTOR, and p‐ERK levels in melanoma cells (Figure 3B). Additionally, downregulation of the Akt pathway by the small molecule inhibitor MK2206 attenuated the activity of magnolol and melanoma cells survived (Figure 3C). Importantly, blocking of the Akt pathway led to reactivation of p‐ERK upon exposure to magnolol which might result in survival of melanoma cells (Figure 3D). This suggests that Akt signaling might play one of the key roles for magnolol‐induced cell death in melanoma.

A previous study reported that PI3K/Akt signaling regulates the active histone mark H4K4me3 by KDM5A phosphorylation in breast cancer.^18^ Thus, we hypothesized that magnolol modulates Akt target genes through histone modifications resulting in apoptosis. A consistent decrease of the H3K4me3 mark was found upon exposure to magnolol compared to DMSO control in melanoma lines. In contrast, Akt pathway activation by SC79 increased H3K4me3 and the combination of SC79 and magnolol increased H3K4me3 in both *BRAF‐* and *NRAS*‐mutant melanoma cells (Figure 3E, Supplem). Magnolol was found to induce DNA fragmentation in a dose‐dependent manner in non‐small cell lung cancer cells.^19^ Along this line, several studies revealed that ɣ‐H2AX and the repressive mark, H3K9me3, deposited at DNA damage sites are considered as markers for the DNA damage response.^20^ Likewise, we have observed an increase of the repressive histone mark H3K9me3 upon magnolol treatment accompanying by upregulation of ɣ‐H2AX. However, Akt activation by SC79 and SC79/magnolol combination rescued the DNA damage induced by magnolol with concomitant loss of H3K9me3 and the ɣ‐H2AX mark (Figure 3E and Figure S2B,C).

**Figure 4:**
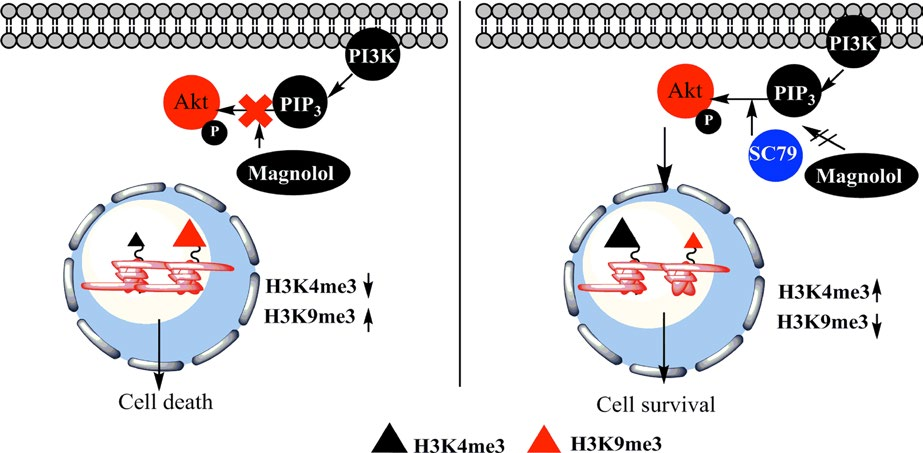
A mechanistic model for the effect of magnolol. Magnolol leads to downregulation of PI3K/Akt signaling in melanoma cells, which results in histone reprogramming with a decrease of the active histone mark H3K4me3 and increase of the repressive histone mark H3K9me3 (left panel). The Akt activator SC79 overcomes the magnolol‐induced inhibition of PI3K/Akt signaling as well as histone reprogramming which leads to cell survival (right panel)

Magnolol‐induced histone remodeling by modulating the PI3K/Akt pathway was further investigated with combination of molecular targeted therapy such as the BRAF/MEK inhibitors dabrafenib, trametinib, and docetaxel. Similar to single exposure with magnolol, combination treatment of magnolol with targeted and chemotherapy resulted in downregulation of H3K4me3 and upregulation of H3K9me3 in melanoma cells. This reciprocal effect on histone modifications was reversible upon activation of the Akt signaling cascade and thus resembling the earlier findings with magnolol alone (Figure 3F, Figure S2D). To summarize magnolol‐induced histone reprogramming characterized by the reduced H3K4me3 mark is indicative of low transcriptional activity and can be salvaged by reactivation of Akt suggestive a plausible role of PI3K/Akt signaling in magnolol‐induced epigenetic modulation and cell death (Figure 4).

## 4 DISCUSSION

Melanoma is considered the deadliest form of skin cancer. Current treatment with targeted therapy shows an initial response, but later resistance develops through intrinsic factors such as activation of mesenchymal, angiogenesis‐related genes^21^ and by acquired mechanisms such as reactivation of the MAPK pathway or activation of alternative prosurvival signaling cascades.^22^ The present study aimed at determining the proposed therapeutic efficacy of a plant‐derived lead compound, magnolol, on melanoma cells either alone or in combination with targeted therapies or chemotherapy.

Magnolol, three derivatives of magnolol and honokiol were initially tested on *NRAS‐* and *BRAF*‐mutant melanoma cells with the aim to identify whether any of the derivatives showed an improved efficacy over the original compound. Out of three compounds, only 5,5'‐di‐(tert‐butyl)‐biphenyl‐2,2'‐diol, a derivative of magnolol, exhibited potent activity against melanoma cells. A recent study identified that 2‐*O*‐methylmagnolol (MM1) was more effective to kill melanoma and squamous carcinoma cells in vitro and in vivo compared to magnolol.^23^ However, our findings suggest that tert‐butyl magnonol was not superior to magnolol in melanoma.

Concentrations down to 30 µmol L^−1^ magnolol‐induced apoptosis and cell death in *NRAS‐* and *BRAF‐*mutant melanoma cells, whereas *BRAF/NRAS* wild‐type melanoma cells were only susceptible at higher concentrations (80 µmol L^−1^). Immortalized keratinocytes were insensitive to magnolol, even at higher concentrations suggesting that magnolol might be more effective in cancer cells. Melanoma cells exhibited G1 phase cell cycle arrest in a concentration‐ and time‐dependent manner. This is in line with a previous finding where magnolol‐induced G0/G1 arrest in gallbladder cancer cells.^24^ Moreover, magnolol‐induced G1 arrest in melanoma spheroids, which resemble the *in vivo* tumor architecture.^13^, ^14^

We found that magnolol downregulates the MAPK‐ERK and PI3K/Akt pathways in a time‐ and dose‐dependent manner. Similar effects were also observed in the 3D spheroid model. An earlier study reported that magnolol downregulates ERK and Akt phosphorylation, albeit at a higher concentration, in non‐small cell lung cancer cells.^19^ However, magnolol did not induce any alteration of the pathways in *BRAF/NRAS* wild‐type melanoma cells and keratinocytes at low concentrations suggestive that magnolol‐induced downregulation of survival pathways might be dependent on the mutation status of cancer cells.

Magnolol was further tested in combination with targeted therapy and chemotherapy. Interestingly, magnolol exhibited a synergistic effect, where it killed melanoma cells at much lower doses of dabrafenib and docetaxel than those currently used in the clinics.^25^ Combined treatment also led to downregulation of the MAPK‐ERK and PI3K/Akt pathways. Our data suggest that magnolol can be used in combination with standard of care targeted therapies for melanoma. Magnolol‐induced cell death has been observed in two melanoma cell lines, A375‐S2 and A431, but at a high concentration (100 µmol L^−1^).^11^ In contrast, we have found that 30 µmol L^−1^ magnolol in monotherapy and 25 µmol L^−1^ in combination therapy were sufficient to induce cell death in *BRAF‐* and *NRAS*‐mutant melanoma cells. Another study demonstrated a potent antitumor effect of honokiol bis‐dichloroacetate in vemurafenib‐resistant melanoma in vivo.^26^ Consistently, a recent study showed a synergistic effect of honokiol and MAPK inhibitor in *BRAFmt* melanoma cells by disrupting mitochondrial electron transport chain.^27^ Since magnolol is structurally similar to honokiol, it is expected to have a similar effect on the *BRAF* inhibitor resistance melanoma cells; however, this requires further investigation.

We then investigated the mechanism of action on PI3K/Akt signaling, rather than MAPK/ERK, as PI3K/AKT signaling is frequently activated as a resistance mechanism in *BRAF*‐mutant melanoma under BRAF/MEK inhibition.^22^ Our findings suggest that activation of the Akt pathway by a small molecule activator rescues the effect of magnolol by increasing PI3K/Akt signaling. Interestingly, this rescue also resulted in reactivation of MAPK‐ERK signaling. Alternatively, blocking of Akt signaling by a small molecule inhibitor led to reactivation of ERK signaling resulting in survival of melanoma cells upon magnolol treatment. A previous study suggests that Akt can suppress Raf kinase by phosphorylation of Ser‐295, which leads to downregulation of MAPK‐ERK signaling.^28^ Therefore, downregulation of Akt signaling might alleviate the repression on Raf kinase which consequently activates ERK signaling.

Magnolol also leads to increased apoptosis by upregulation of caspase‐3 either alone or in combination with targeted‐ and chemotherapy. Indeed, it has been reported that magnolol upregulates apoptotic proteins like caspases‐8,9, cleaved caspase‐3, PARP and reciprocally downregulate anti‐apoptotic proteins such as Bcl‐2 and Mcl‐1.^19^, ^24^ Moreover, PI3K/Akt signaling is known to up‐regulate anti‐apoptotic proteins like Bcl‐2 and Mcl‐1 thus promoting cancer cell survival.^29^ Therefore, it can be inferred that magnolol‐induced downregulation of PI3K/Akt signaling might also deregulate the balance of anti‐apoptotic and apoptotic proteins resulting in melanoma cell death.

Although some of the earlier findings reported the effect of magnolol on multiple signaling cascades including PI3K/Akt,^17^, ^19^ it is unknown whether the downregulation of the PI3K/Akt pathway might have any consequences on transcriptional changes of genes through epigenetic modifications.

To the best of our knowledge, we found for the first time that both *BRAF*‐ and *NRAS*‐mutant melanoma cells exposed to magnolol exhibited lower levels of the active histone mark H3K4me3, which presumably will lead to less transcriptional activity. The magnolol‐induced decrease of H3K4me3 was salvaged by an Akt activator, which was also true for combined targeted‐ and chemotherapy. Similarly, this combinatorial effect on histone marks was rescued by activating the Akt pathway. A previous study reported that PI3K/Akt signaling regulates the H3K4me3 mark through KDM5A phosphorylation in breast cancer.^18^ Phospho‐Akt can prevent nuclear localization of KDM5A by inducing phosphorylation of KDM5A. Since KDM5A is a demethylase of H3K4me3, preventing nuclear localization of KDM5A by Akt downregulation led to an increase of H3K4me3.^18^ Likewise, we have observed that the downregulation of PI3K/Akt by magnolol led to a decrease of H3K4me3. Therefore, we speculate that by downregulating p‐Akt, magnolol might also modulate KDM5A and thus regulate gene expression through H3K4me3.

Conversely, the increase of the repressive histone mark, H3K9me3 was consistently observed in *BRAF‐* and *NRAS‐*mutant melanoma cells upon exposure to magnolol and decreased upon activation of Akt. Moreover, we also observed the increase of the DNA damage marker ɣ‐H2AX in the magnolol‐treated cell lines. This supports previous findings, where magnolol has been reported to induce DNA damage in gastric adeno‐carcinoma cells^17^ and DNA damage has been also reported to induce the H3K9me3 mark.^20^

These accumulative findings suggest that magnolol is a potential therapeutic option for treating *BRAF*‐mutant metastatic melanoma in combination with current targeted therapies. Combined magnolol/dabrafenib/trametinib potentiates a synergistic effect by significantly reducing the dosage of monotherapies. The presence of a nonsignaling driver mutation (due to targeted therapy) in the presence of magnolol might confer increased susceptibility. By reducing the dosage of both targeted therapies and magnolol, patients may experience a better outcome with less side effects and delayed relapse. An important limitation of this study is to test the combination of magnolol and MAPK inhibitor in vivo. Therefore, a dose escalating pre‐clinical study should be performed in the future. This study also highly demands a comprehensive ChIP‐seq analysis of H3K4me3 and H3K9me3 to decipher underlying downstream epigenetic targets of H3K4me3 and their functional relevance on cell death upon treatment with magnolol compared to untreated control.

## CONFLICT OF INTEREST

The authors declare no conflict of interest.
