# Supplementary figures and images for: Magnolol induces cell death through PI3K/Akt‐mediated epigenetic modifications boosting treatment of BRAF‐ and NRAS‐mutant melanoma
Source: Cancer Med. 2019 Feb 21;8(3):1186–96. doi: 10.1002/cam4.1978 (PMC6434221; doi:10.1002/cam4.1978)

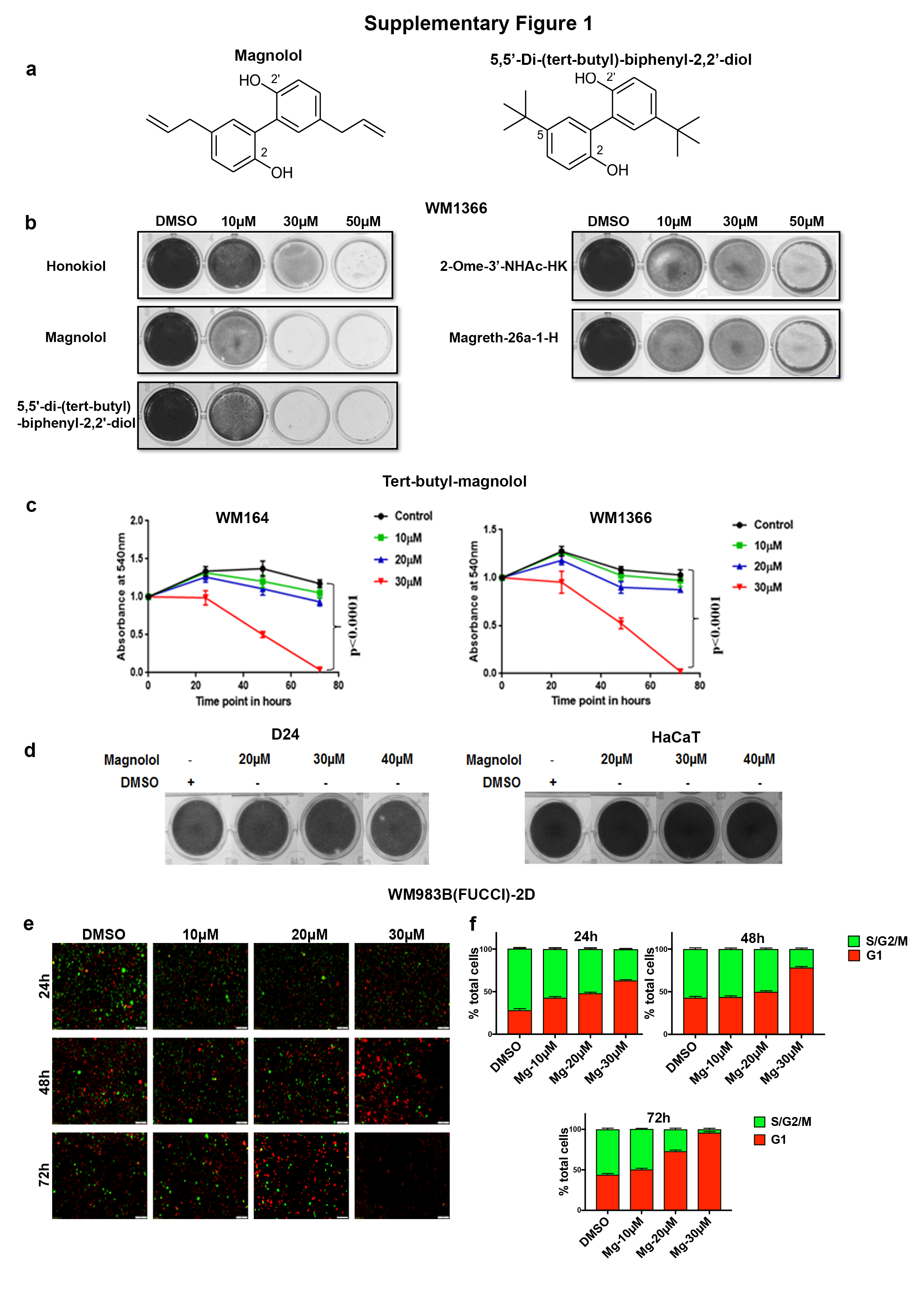

Supplement: Supplementary file 1 [file CAM4-8-1186-s001.tif]

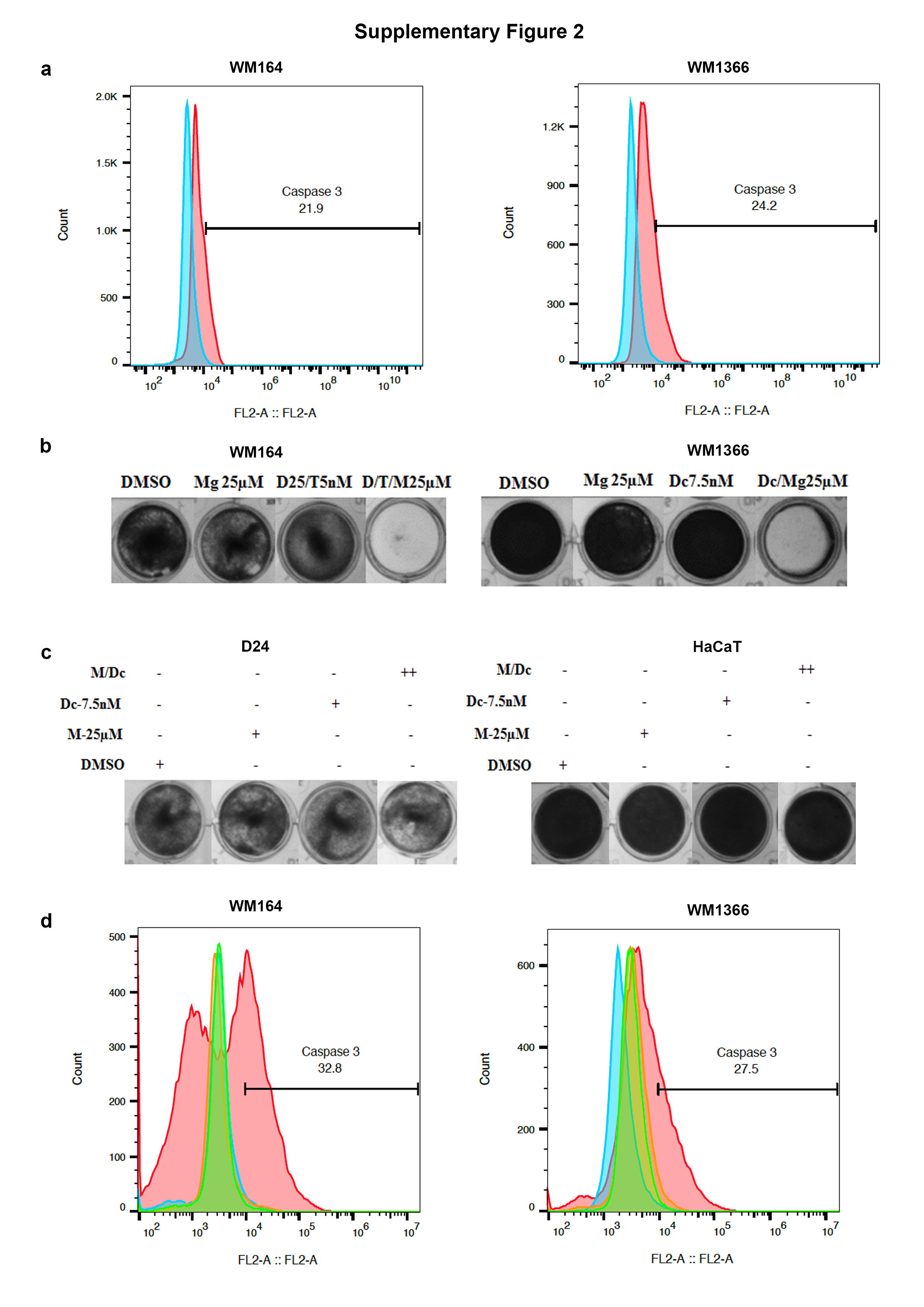

Supplement: Supplementary file 2 [file CAM4-8-1186-s002.tif]

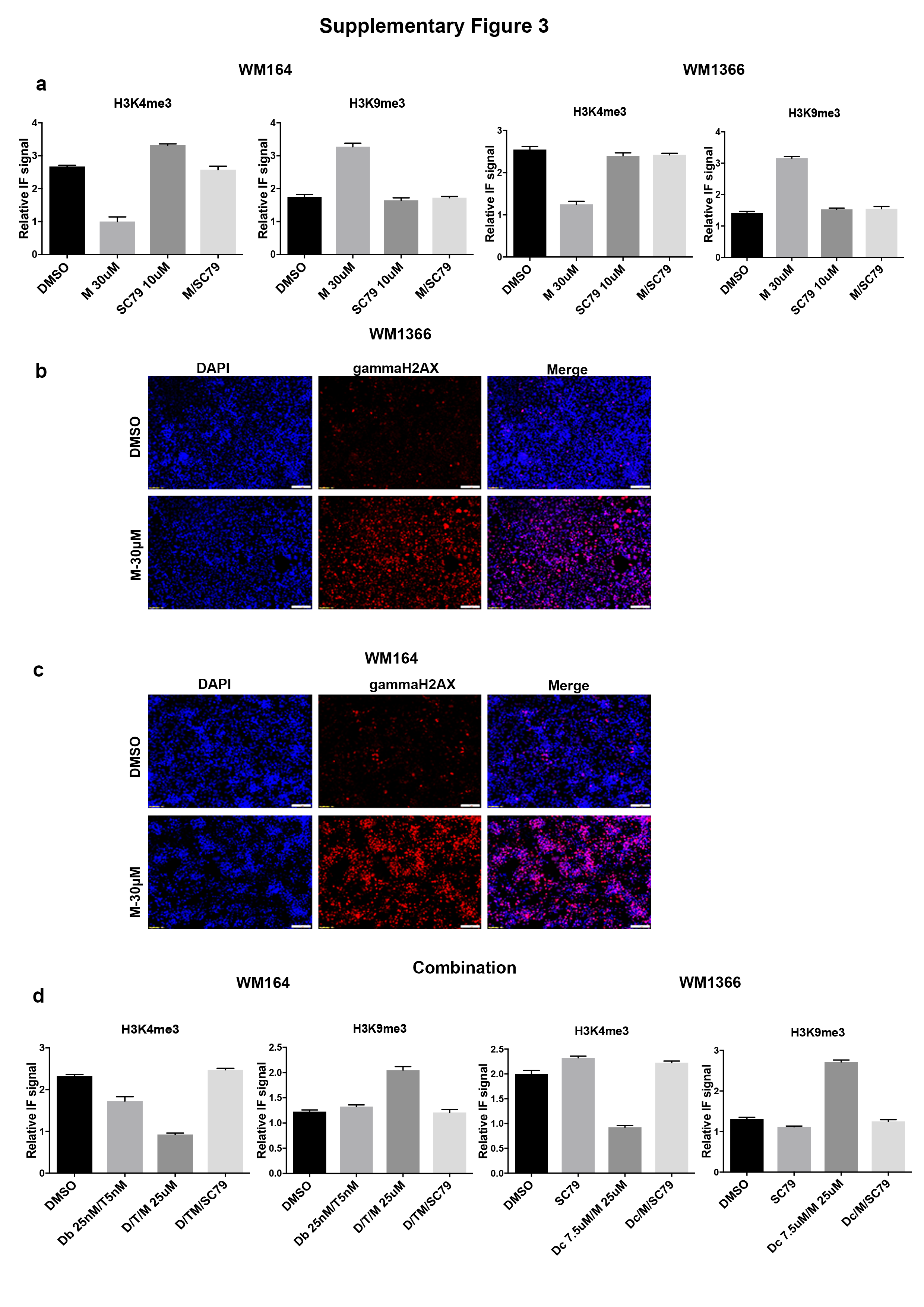

Supplement: Supplementary file 3 [file CAM4-8-1186-s003.tif]
