# Supplementary material for: Magnolol induces cell death through PI3K/Akt‐mediated epigenetic modifications boosting treatment of BRAF‐ and NRAS‐mutant melanoma
Source: Cancer Med. 2019 Feb 21;8(3):1186–96. doi: 10.1002/cam4.1978 (PMC6434221; doi:10.1002/cam4.1978)
Supplement: Supplementary file 4 [file CAM4-8-1186-s004.docx]

**Supplementary Figures and Table:**

**Supplementary Figure S1 (Related to Figure 1): Effect of 5,5'-di-(*tert*-butyl)-biphenyl-2,2'-diol and magnolol on melanoma cells and keratinocytes. (a)** Structures of magnolol and 5,5'-di-(*tert*-butyl)-biphenyl-2,2'-diol. **(b)** WM1366 cells were treated with either DMSO or increasing dosage of the indicated compounds for 72h followed by crystal violet staining. **(c)** WM164 and WM1366 cells were treated with either DMSO or an increasing concentration of tert-butyl magnonol as indicated. Cell survival assay was performed by MTT assay after treatment over 24, 48 and 72 h. Statistical analysis was performed by one-way ANOVA test. **(d)** D24 or HaCaT cells were treated with either DMSO or magnolol at indicated doses for 72 h followed by crystal violet staining. **(e)** Epifluorescence microscopy images of FUCCI-WM983b cells in 2D culture after treatment with magnolol with indicated concentrations and treatment periods. DMSO was used as control. Red, G1-phase; yellow, early S-phase; green, late S/G2/M-phase. **(f)** Quantification of the FUCCI red and green images by ImageJ, n=2 independent experiments

**Supplementary Figure S2 (Related to Figure 1,2): Synergistic effect of magnolol and targeted/chemotherapy in melanoma cells. (a)** WM164 and WM1366 cells were treated with DMSO and 30 µM magnolol for 60 h. Cells were fixed with 4% PFA following treatment and stained with caspase-3 antibody. Representative (n=3) histograms (FL2-A) from one biological replicate each for caspase-3. The blue peak represents the DMSO control and the red peak magnolol-treated cells. **(b)** WM164 cells were treated with DMSO, 25 µM magnolol, 25 nM dabrafenib/5 nM trametinib or 25 µM Mg/25 nM dabrafenib/5 nM trametinib for 72 h. Whereas WM1366 cells were exposed to DMSO, 25 µM Mg, 7.5 nM docetaxel or 25 µM magnolol/7.5 nM docetaxel for 72 h. Cells were fixed with 4% PFA and stained with 0.5% crystal violet (n=3, biological replicates). **(c)** D24 and HaCaT cells were treated with 25 µM Mg, 7.5 nM docetaxel for 72 h and stained similarly with crystal violet. **(d)** WM164 cells treated DMSO, 25 µM Mg, 25 nM dabrafenib/5nM trametinib or 25 µM magnolol/25 nM dabrafenib/5 nM trametinib whereas WM1366 cells were treated with DMSO, 25 µM magnolol, 7.5nM docetaxel or 25 µM magnolol/7.5 nM docetaxel for 60 h. Cells were fixed with 4% PFA following treatment and stained with caspase-3 antibody. Representative (n=3) histograms (FL2-A) from one biological replicate each for caspase-3. Green, orange, blue and red peaks represent DMSO control, dab/tra, Mg and dab/tra/Mg, respectively (WM164). Similarly, blue, orange, green and red peaks represent DMSO control, Mg, doce and doce/Mg respectively (WM1366).

**Supplementary Figure S3 (Related to Figure 3): Magnolol leads to histone modifications and an increase of the γH2AX. (a)** Immunofluorescence images of the representative histone marks have been quantified by ImageJ and plotted in graphpad prism, n=2 independent experiments. **(b)** WM1366 and WM164 cells **(c)** were treated with either DMSO or 30 µM magnolol for 48 h. Cells were then stained with a γH2AX antibody and expression of this mark was assessed by immunofluorescence. Nuclear staining (DAPI, blue) was merged with the indicated antibody (red) (10× magnification). **(d)** Similar to (a) histone marks were quantified by ImageJ.

Supplementary Table1: Chemical structures of different derivatives of honokiol including honokiol along with their chemical name, molecular weight and mass^[^[^1^](#_ENREF_1)^]^.

| **Number** | **Formula** | **Code** | **Molecular Weight** | **Amount (mg)** |
| --- | --- | --- | --- | --- |
| 1 | 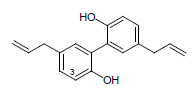 | Magnolol | 266 | 1.2 |
| 2 | 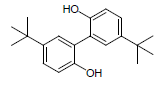 | Tert-butyl-magnolol Magreth-33 | 298 | 1.0 |
| 3 | 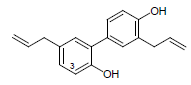 | Honokiol | 266 | 1.7 |
| 4 | 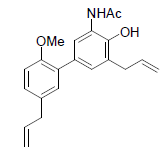 | 2-OMe-3’-NHAc-HK(“PMB_100A”) | 337 | 1.0 |
| 5 | 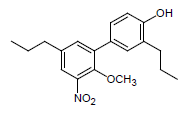 | Magreth-26a-1-H (108A) | 329 | 1.0 |

**Reference:**

1. Marketa Bernaskova, N.K., Wolfgang Schuehly, Antje Huefner, Robert Weis and Rudolf Bauer, *Synthesis of tetrahydrohonokiol derivatives and their evaluation for cytotoxic activity against CCRF-CEM Leukemia, U251 Glioblastoma and HCT-116 colon cancer cells.* Molecules, 2014. **19**: p. 1223-1237.
