# Supplementary material for: Magnolol induces cell death through PI3K/Akt‐mediated epigenetic modifications boosting treatment of BRAF‐ and NRAS‐mutant melanoma
Source: Cancer Med. 2019 Feb 21;8(3):1186–96. doi: 10.1002/cam4.1978 (PMC6434221; doi:10.1002/cam4.1978)
Supplement: Supplementary file 5 [file CAM4-8-1186-s005.docx]

**Materials and Methods:**

**Antibodies and Reagents:** p-mTOR, t-mTOR and actin antibodies were purchased from Abcam (CBG, UK). Phosphorylated AKT–Ser473 (p-AKT), total AKT (t-AKT), phosphorylated ERK1,2-Thr202/Tyr204 (p-ERK1,2), total ERK (ERK1,2) antibodies were obtained from Cell Signaling Technology (MA, USA). Antibodies for H3K4me3, H3K9me3 were purchased from Active Motif (CA, USA). Alexa Fluor 594 antibody and Hoechst 33342 were obtained from Thermo Fisher Scientific (MA, USA) and the anti-gammaH2AX antibody from Abcam (CBG, UK)

**Drugs:** Docetaxel, dabrafenib, trametinib, honokiol, magnolol, Akt activator (SC79) and pan-Akt inhibitor (MK2206) were purchased from Selleck Biochem, TX, USA. The synthesis of 5,5'-di-(*tert*-butyl)-biphenyl-2,2'-diol, 2-Ome-3’-NHAc-HK and Magreth-26a-1-H (in Supplemental Table 1) was done by Dr. Antje Huefner at the University of Graz, Austria.

**Cell lines**: Melanoma cell lines WM164, WM1366 and HaCaT were obtained from The Wistar Institute. D24 melanoma cell line was a generous gift from Professor Brian Gabrielli, Mater Research Institute, The University of Queensland. Each cell line was authenticated by STR fingerprinting (Garvan Molecular Genetics Facility, Sydney, NSW, Australia) and tested for mycoplasma contamination.

**Cell culture and drug treatment:** Cells were cultured in RPMI medium (Life technology, USA) supplemented with 5% heat-inactivated fetal bovine serum (FBS, Sigma, MI), 10% of L-Glutamine and 10% of penicillin-streptomycin antibiotics (Life Technologies, CA). WM164, WM1366, D24 and HaCaT cells were treated with increasing concentrations of magnolol and derivatives (0-50µM) for 24, 48 and 72 hours separately. For the combination studies, melanoma cells were exposed to DMSO, magnolol, dabrafenib/trametinib, magnolol/dabrafenib/trametinib, docetaxel or magnolol/docetaxel. All experiments were performed in biological triplicates.

**Crystal violet staining**: Drug-treated melanoma cells and the corresponding parental cells were washed with 1xPBS and then fixed with 4% paraformaldehyde, followed by 30 min incubation in room temperature. Fixed cells were incubated with 0.5% crystal violet solution in 1xPBS for one hour. The plates were then washed three times with RO H_2_O and pictures were taken using the Bio Rad gel documentation unit after drying.

**Cell survival assay:** Cell viability was analysed by MTT assay. Briefly, 2x10^4^ cells were seeded in 96-well culture plates. On the following day cells were exposed to the drugs as indicated above and after 72 h of drug exposure cells were incubated with MTT (3-(4, 5-dimethylthiazolyl-2)-2,5 diphenyltetrazolium bromide) reagent (Life Technologies, CA) (10 µl) at 37ºC for 4 h. 100 µl detergent reagent was then added to the wells and incubated in the dark at room temperature for 4 h. Absorbance was measured at 570 nm using a microtiter plate reader.

**Caspase-3 apoptosis assay:** The caspase-3 apoptosis assay was carried out using a PE Active Caspase-3 Apoptosis Kit™ from BD Pharmingen, according to the manufacturer’s protocol. The cells were washed and permeabilized with cytofix/cytoperm solution followed by washing and re-incubation with the caspase-3 antibody further analyzed by flow cytometry. All experiments were done in biological triplicate.

**Cell lysates and Immunoblots:** Immunoblotting was performed as previously described^1^. Briefly, cell pellets were lysed with RIPA buffer which enables efficient cell lysis for protein isolation while avoiding protein degradation. Protein samples were treated with protease and phosphatase inhibitor cocktail (Cell Signaling Technology, MA, USA) before immunoblotting. Protein concentrations were determined by BCA standard solution. Equal amounts of protein were loaded onto 10% SDS-PAGE gels. Polyvinylidene difluoride (PVDF) membrane was used for wet transfer. Transferred proteins were blocked with either 5% BSA (for phosphorylated proteins) or 5% milk in 1xTBST for one hour at room temperature. The membrane was then incubated with a specific primary antibody overnight at 4^o^C, then washed three times with 1xTBST and incubated with a secondary antibody at room temperature for 1hr followed by three washes with 1xTBST. Membrane was developed with ECL solution and image taken with LI-COR C-DiGiT Western blot scanner.

**Immunofluorescence:** Immunofluorescence (IF) analysis was performed as described previously^2^. Briefly, treated or untreated cells were fixed with 4% paraformaldehyde in PBS for 30 minutes and then blocked with 0.3% Triton X-100, 5% goat serum and 1% BSA dissolved in PBS. Cells were incubated with the primary antibody at 4^o^C overnight; followed by three washes with 1% BSA in PBS on the following day. Cells were incubated with Alexa fluoro 594 secondary antibody for one hour along with Hoechst dye for the nuclear staining. All images were taken with an Olympus inverted fluorescent microscope.

**3D melanoma spheroid model:** Melanoma spheroids were prepared as described^3, 4^. Briefly, 100 μl of 1.5% agarose dissolved in PBS were poured onto each well of a 96-well plate and incubated at room temperature for one hour to solidify. Afterwards, 200 μl of WM164F melanoma cells (25,000 cells/ml) were seeded on top of the solid agar. 96-well plates were incubated for 72 h at 37˚C before drug treatment.

**Spheroid treatment:** After formation of 3D spheroids, morphology was checked using bright field microscopy. Only properly formed spheroids were further selected for drug treatment. Briefly, 100 μl of medium were carefully aspirated from the top of spheroid suspensions. Spheroid plates were refilled with 100 μl of medium containing either DMSO or 30 μM magnolol. Treated spheroids were incubated for 48 h. After 48 h, cell cycle images were taken using epifluorescence microscopy followed by sample collection for protein isolation.

**Fluorescent ubiquitination-based cell cycle indicator (FUCCI):** To generate stable melanoma cell lines expressing the FUCCI constructs, mKO2-hCdt1 (30-120) and mAG-hGem (1-110)^5^ were subcloned into a replication-defective, self-inactivating lentiviral expression vector system as previously described^6^. The lentivirus was produced by co-transfection of human embryonic kidney 293T cells. High-titer viral solutions for mKO2-hCdt1 (30/120) and mAG-hGem (1/110) were prepared and used for co-transduction into eight biologically and genetically well-characterized melanoma cell lines (see above) and subclones were generated by single cell sorting^4, 7, 8^. Images were captured using epifluorescence microscopy and signals were quantified using ImageJ software.

**References:**

1. Ravindran Menon D, Das S, Krepler C, Vultur A, Rinner B, Schauer S, Kashofer K, Wagner K, Zhang G, Bonyadi Rad E, Haass NK, Soyer HP, et al. A stress-induced early innate response causes multidrug tolerance in melanoma. *Oncogene* 2015;**34**: 4448-59.

2. Al Emran A, Marzese DM, Menon DR, Stark MS, Torrano J, Hammerlindl H, Zhang G, Brafford P, Salomon MP, Nelson N, Hammerlindl S, Gupta D, et al. Distinct histone modifications denote early stress-induced drug tolerance in cancer. *Oncotarget* 2018;**9**: 8206-22.

3. Smalley KS, Lioni M, Noma K, Haass NK, Herlyn M. In vitro three-dimensional tumor microenvironment models for anticancer drug discovery. *Expert Opin Drug Discov* 2008;**3**: 1-10.

4. Spoerri L, Beaumont KA, Anfosso A, Haass NK. Real-Time Cell Cycle Imaging in a 3D Cell Culture Model of Melanoma. *Methods Mol Biol* 2017;**1612**: 401-16.

5. Sakaue-Sawano A, Kurokawa H, Morimura T, Hanyu A, Hama H, Osawa H, Kashiwagi S, Fukami K, Miyata T, Miyoshi H, Imamura T, Ogawa M, et al. Visualizing spatiotemporal dynamics of multicellular cell-cycle progression. *Cell* 2008;**132**: 487-98.

6. Smalley KS, Brafford P, Haass NK, Brandner JM, Brown E, Herlyn M. Up-regulated expression of zonula occludens protein-1 in human melanoma associates with N-cadherin and contributes to invasion and adhesion. *Am J Pathol* 2005;**166**: 1541-54.

7. Haass NK, Beaumont KA, Hill DS, Anfosso A, Mrass P, Munoz MA, Kinjyo I, Weninger W. Real-time cell cycle imaging during melanoma growth, invasion, and drug response. *Pigment Cell Melanoma Res* 2014;**27**: 764-76.

8. Beaumont KA, Anfosso A, Ahmed F, Weninger W, Haass NK. Imaging- and Flow Cytometry-based Analysis of Cell Position and the Cell Cycle in 3D Melanoma Spheroids. *J Vis Exp* 2015;**106**: e53486.
